# Mesenchymal stromal cell-mediated mitochondrial transfer unveils new frontiers in disease therapy

**DOI:** 10.1186/s13287-025-04675-x

**Published:** 2025-10-08

**Authors:** Huan Chen, Xu Chen, Zi-hao Zhou, Jia-rong Zheng, Ye Lu, Pei Lin, Yun-fan Lin, Yu-cheng Zheng, Bin Xiong, Rong-wei Xu, Li Cui, Xin-yuan Zhao

**Affiliations:** 1 School of Stomatology, Stomatological Hospital, Southern Medical University, Guangzhou, 510280 Guangdong China; 2 Department of Dentistry, The First Affiliated Hospital, Sun Yat-Sen University, Guangzhou, 510080 China; 3 School of Dentistry, University of California, Los Angeles, Los Angeles, CA 90095 USA

**Keywords:** Mesenchymal stromal cell, Mitochondrial transfer, Therapeutic efficacy

## Abstract

Mitochondrial dysfunction is a pivotal factor in the progression of various diseases, making it a critical therapeutic target. Mesenchymal stromal cells (MSCs) have shown promise in mitigating this dysfunction through the transfer of healthy mitochondria to damaged cells. This review comprehensively analyzes the mechanisms of MSC-derived mitochondrial transfer, including tunneling nanotubes (TNTs) and extracellular vesicles, and highlights their therapeutic potential across a spectrum of diseases, such as neurodegenerative disorders, ocular diseases, and inflammatory conditions. Additionally, strategies to enhance mitochondrial transfer efficiency—such as genetic modifications and optimization of MSC sources—are thoroughly explored. Despite these promising findings, challenges remain, including the need for a deeper understanding of transfer mechanisms, ensuring the quality and functionality of transferred mitochondria, and addressing potential immune responses. While MSC-derived mitochondrial transfer holds significant therapeutic potential, careful consideration of its dual nature, especially in specific pathological contexts such as cancer, is essential. With further research and technological advancements, this approach could become a cornerstone in the treatment of diseases characterized by mitochondrial dysfunction.

## Background

**Fig. 1:**
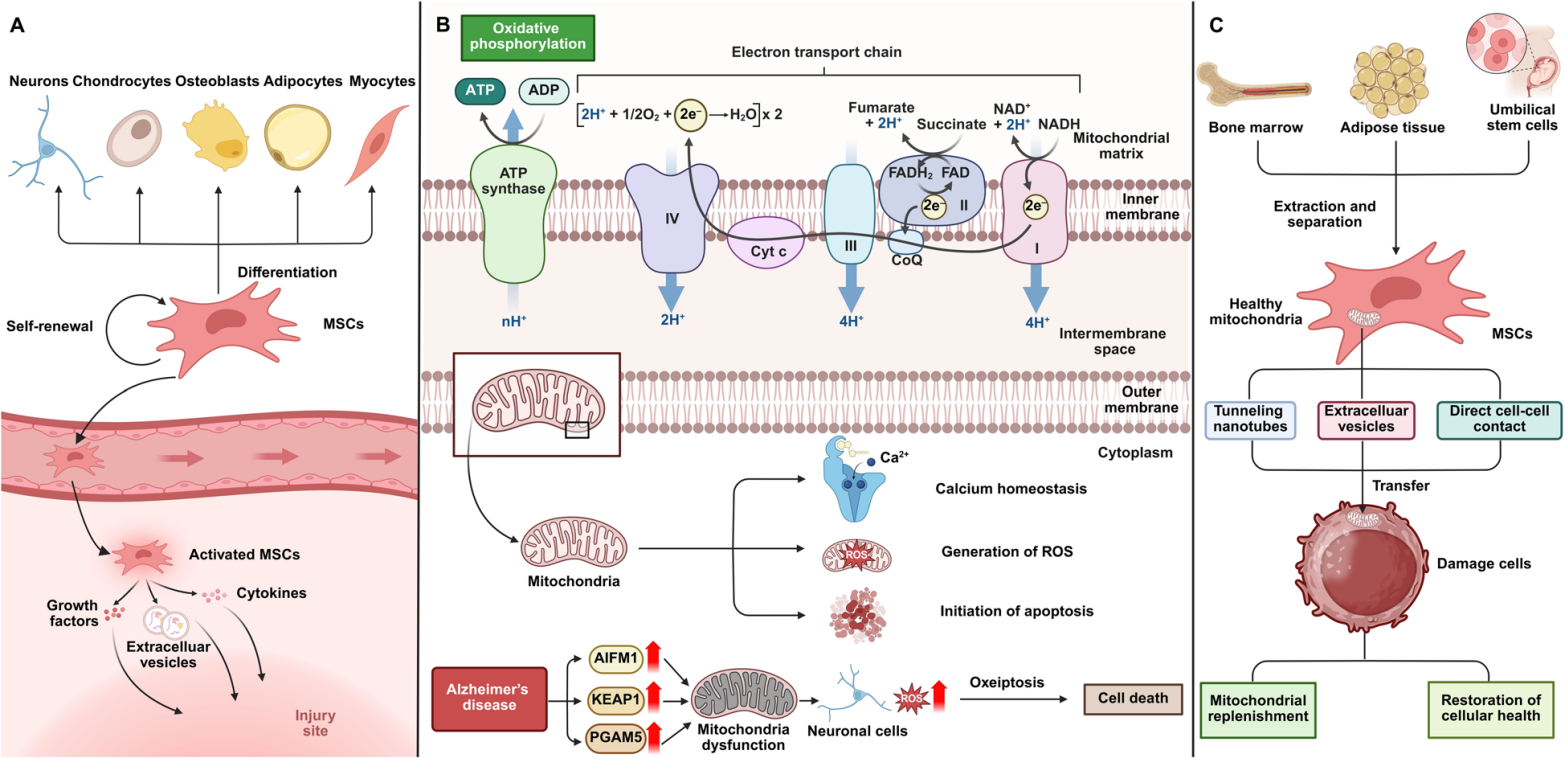
MSC-mediated differentiation, mitochondrial function, and therapeutic mitochondrial transfer for cellular repair. **A** MSCs can self-renew and differentiate into various cell types, including neurons, osteoblasts, and adipocytes, in response to environmental signals. After injury, activated MSCs migrate to the damage site, release growth factors and cytokines, and secrete extracellular vesicles to modulate the microenvironment for tissue repair [1, 2]. **B** The electron transport chain (ETC) in mitochondria drives oxidative phosphorylation, generating ATP. Mitochondrial dysfunction leads to ROS production, calcium imbalance, and apoptosis. In diseases like Alzheimer’s, oxeiptosis and mitochondrial failure contribute to neuronal damage [5–7]. **C** MSCs from bone marrow or adipose tissue can transfer healthy mitochondria to damaged cells via TNTs, extracellular vesicles, or direct contact, promoting mitochondrial replenishment and restoring cellular function [8]

MSCs are multipotent stromal cells found in various mesenchymal tissues, including adipose tissue, umbilical cord, and bone marrow. These cells possess the remarkable ability to differentiate into multiple cell types, such as osteoblasts, chondrocytes, and adipocytes, making them essential players in tissue repair and regeneration [1, 2]. In addition to their differentiation potential, MSCs exhibit significant immunomodulatory properties. They migrate to sites of injury, where they secrete a diverse array of cytokines, growth factors, and extracellular vesicles that modulate immune responses and create a supportive environment for tissue healing (Fig. 1A). These combined characteristics position MSCs as highly promising candidates for treating a broad spectrum of diseases, including autoimmune disorders, musculoskeletal injuries, and neurodegenerative conditions [3].

Mitochondria are essential organelles responsible for producing the majority of cellular ATP through oxidative phosphorylation, making them central to energy metabolism and overall cellular function [4]. Beyond their role as the cell’s powerhouse, mitochondria are integral to regulating several key cellular processes, including the maintenance of calcium homeostasis, the generation of reactive oxygen species (ROS), and the initiation of apoptosis [5]. By modulating these functions, mitochondria influence cellular differentiation, signaling pathways, and stress responses, thereby playing a crucial role in maintaining cellular homeostasis and adaptation to environmental changes. Mitochondrial dysfunction is increasingly recognized as a pivotal factor in the progression of various diseases [6]. For instance, mitochondrial dysfunction, marked by elevated levels of KEAP1, PGAM5, and AIFM1, drives neuronal cell death in Alzheimer’s disease (AD) through the oxeiptosis pathway. Interestingly, oxeiptosis is a form of caspase-independent, ROS-induced programmed cell death that is regulated by KEAP1–PGAM5–AIFM1 signaling. It is triggered under conditions of excessive oxidative stress and serves as a protective mechanism to eliminate damaged cells without provoking inflammation [7] (Fig. 1B). Similarly, mitochondrial dysfunction drives disease progression in ischemic myocardial conditions by promoting endothelial-to-mesenchymal transition. Melatonin counters this dysfunction, reducing oxidative stress and preserving endothelial function, thereby mitigating cardiac damage and slowing disease progression [8] (Fig. 1C).

Targeting mitochondria has emerged as a critical strategy for treating diseases, given the central role of mitochondrial dysfunction in the pathogenesis of a wide range of conditions [9]. One promising approach in this context is the use of MSC-derived mitochondrial transfer. This strategy involves transferring healthy mitochondria from MSCs to damaged cells, thereby replenishing functional mitochondria and restoring cellular health. Compared with the direct administration of isolated mitochondria, MSC-mediated mitochondrial transfer offers a series of distinct biological advantages that enhance its therapeutic potential. MSCs possess inherent homing capabilities, allowing them to migrate preferentially toward sites of tissue injury, inflammation, or metabolic stress—thereby ensuring spatially targeted delivery of functional mitochondria to damaged cells [10, 11]. In contrast to the passive nature of isolated mitochondrial therapy, MSCs dynamically sense and respond to microenvironmental cues, enabling them to fine-tune the timing, magnitude, and directionality of mitochondrial transfer in a context-dependent manner [12]. Moreover, mitochondria transferred via MSCs remain within a protected cellular compartment during trafficking, reducing exposure to extracellular stressors and enzymatic degradation, which significantly compromises the efficacy of isolated mitochondria delivered in cell-free systems. Beyond their role as mitochondrial donors, MSCs also exert a wide range of paracrine and immunomodulatory effects that support tissue repair, modulate inflammation, and promote cellular recovery, collectively enhancing therapeutic outcomes [13–15]. These integrated features render MSC-mediated mitochondrial transfer a more robust, regulated, and physiologically responsive strategy for treating diseases associated with mitochondrial dysfunction. In this review, we first provide a systematic and critical analysis of the current evidence supporting the use of MSC-mediated mitochondrial transfer in treating human diseases. Next, strategies to enhance the efficiency of MSC mitochondrial transfer are summarized. Finally, we discuss the challenges and future prospects of employing MSC-derived mitochondrial transfer as a therapeutic approach.

## The role of mitochondrial transfer in regulating cellular function and disease progression

**Fig. 2:**
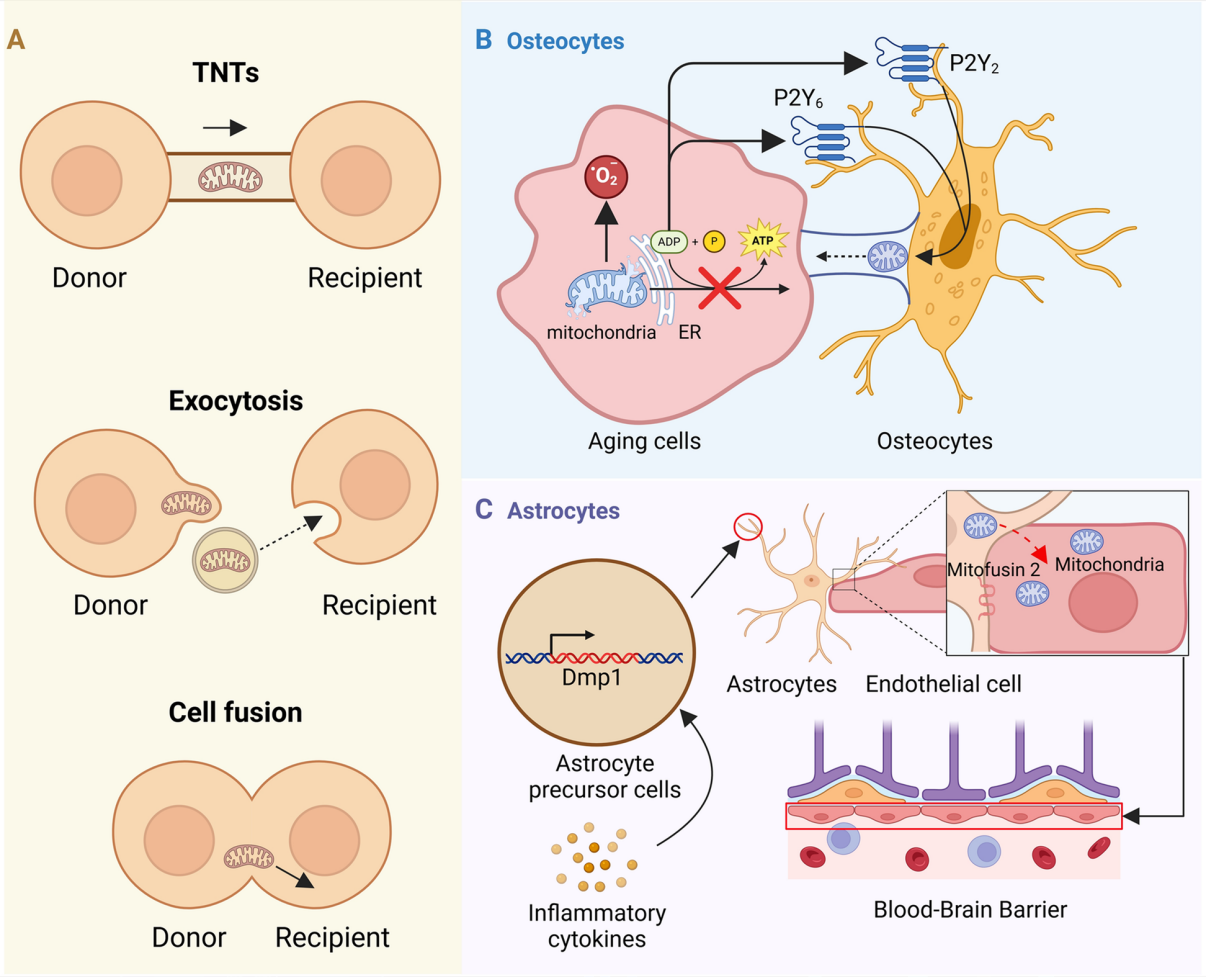
Mitochondrial transfer as a key regulator of cellular function. **A** Three major mechanisms of mitochondrial transfer: TNTs, exocytosis, and cell fusion. These pathways enable mitochondrial exchange between donor and recipient cells, regulating cellular homeostasis [17]. **B** In osteocytes, mitochondrial transfer is triggered by ADP release from stressed cells, activating P2Y₂ and P2Y₆ receptors. This process enhances energy metabolism, reduces oxidative stress, and maintains bone homeostasis [18]. **C** In astrocytes, mitochondria are transferred to endothelial cells through Dmp1-expressing endfeet. Loss of Mitofusin 2 disrupts this process, causing blood–brain barrier (BBB) damage and neuroinflammation [19]

**Fig. 3:**
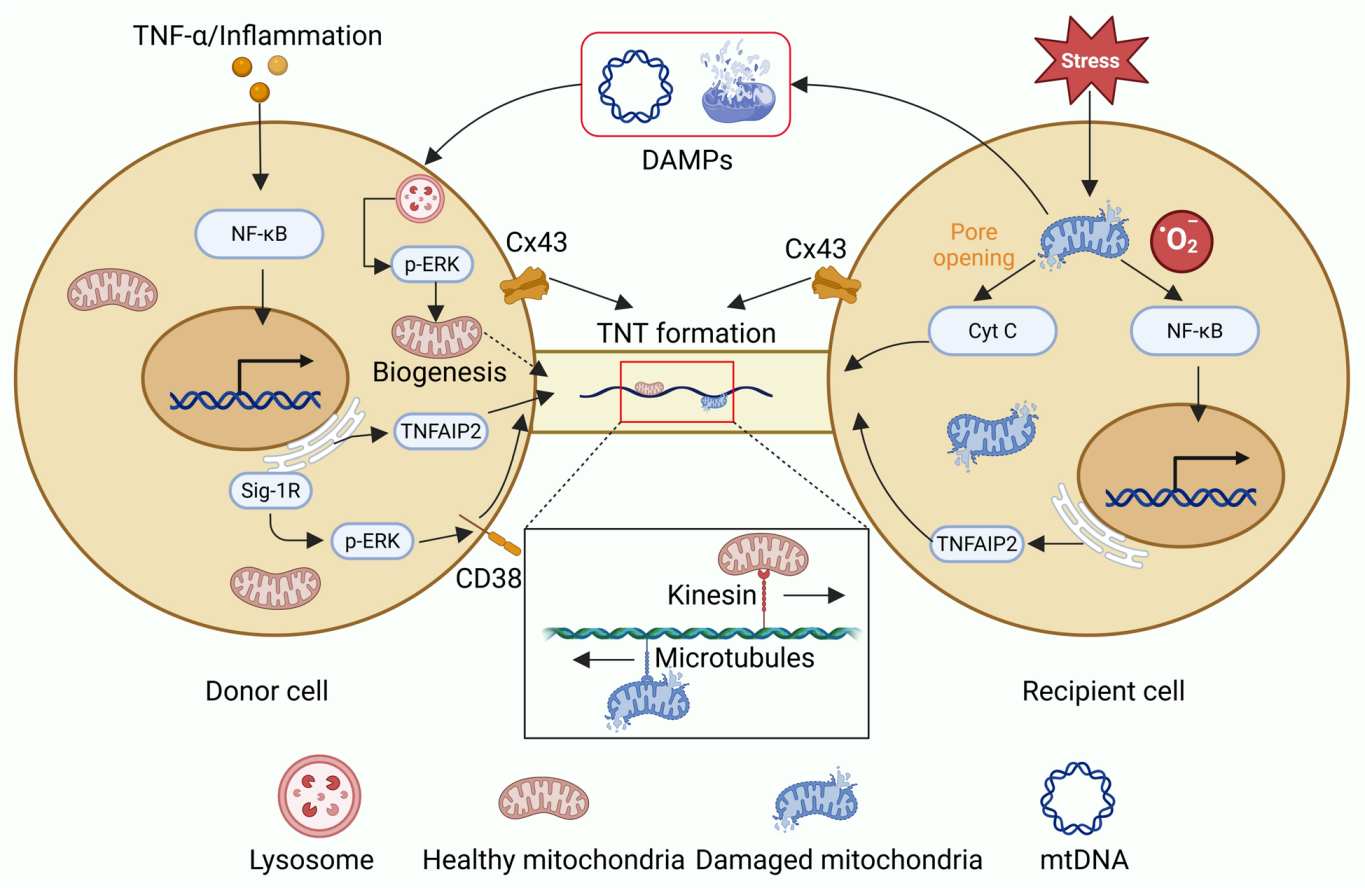
Molecular mechanisms underlying TNT-mediated mitochondrial transfer. Under stress conditions, recipient cells exhibit mitochondrial dysfunction and elevated ROS, leading to NF-κB activation and pro-inflammatory signaling. In response, donor cells initiate mitochondrial biogenesis via NF-κB, p-ERK, CD38, and Sig-1R/TNFAIP2 signaling. TNTs formed through connexin 43 (Cx43) facilitate directional mitochondrial transfer along microtubules via kinesin, promoting mitochondrial restoration and cellular homeostasis [17]

Mitochondrial transfer is a critical cellular process wherein mitochondria are transferred between cells, playing a pivotal role in modulating cellular homeostasis and function [16]. This transfer occurs through three primary mechanisms: TNTs, extracellular vesicles, and direct cell–cell contact. TNTs are dynamic cytoplasmic extensions that connect distant cells, facilitating the direct exchange of healthy mitochondria to damaged cells in response to stress or injury, thereby restoring mitochondrial function and maintaining cellular homeostasis. Extracellular vesicles, including exosomes and microvesicles, act as carriers of mitochondria and mitochondrial components, enabling remote modulation of cellular metabolism and function through their uptake by recipient cells, a process particularly significant in immune responses and tissue repair. Additionally, mitochondria can be transferred through direct cell–cell contact, where adjacent cells form transient or stable junctions, such as gap junctions, to coordinate cellular function in tissues with high metabolic demands. These mechanisms collectively ensure the efficient distribution of mitochondria, enabling cells to adapt to metabolic demands and stress [17] (Figs. 2A, 3).

It has been well documented that mitochondrial transfer plays a critical role in modulating cellular function. For instance, mitochondrial transfer in osteocytes, triggered by ADP release from stressed cells and mediated by P2Y2 and P2Y6 receptors, restores cellular function by enhancing energy metabolism and reducing oxidative stress, particularly in aging [18] (Fig. 2B). Additionally, astrocyte-mediated mitochondrial transfer to ECs via Dmp1-expressing astrocyte endfeet is crucial for maintaining blood–brain barrier (BBB) integrity. Loss of Mitofusin 2 in astrocytes disrupts this transfer, leading to BBB leakage [19] (Fig. 2C). Notably, mitochondrial transfer serves as a double-edged sword in disease modulation. It offers therapeutic potential by restoring mitochondrial function in compromised cells, aiding in disease treatment. For instance, mitochondrial transfer from MSCs to ECs via TNTs enhances EC engraftment under stress, a process essential for forming functional vessels in ischaemic tissues [20]. Similarly, astrocytic LRP1 promotes mitochondrial transfer to neurons by reducing lactate production, protecting against ischemic stroke. Inhibition of LRP1 impairs this transfer, worsening neuronal damage, while elevated lactate in stroke patients correlates with reduced mitochondrial transfer [21].

However, mitochondrial transfer can also facilitate the transfer of dysfunctional mitochondria, potentially amplifying cellular dysfunction and accelerating disease pathogenesis [22, 23]. Mitochondrial transfer from adipose stem cells to breast cancer cells via TNTs enhances ATP production, driving multi-drug resistance (MDR) through oxidative phosphorylation. Blocking this transfer reduces HIF-1α expression and MDR, suggesting a potential therapeutic strategy for breast cancer treatment by targeting mitochondrial transfer [24]. Additionally, extracellular vesicles transfer 3R and 4R tau from neurons to astrocytes, disrupting mitochondrial function and contributing to disease progression. This extracellular vesicle-mediated mitochondrial dysfunction highlights a key mechanism in the spread of tauopathies and neurodegeneration [25].

## MSC-derived mitochondrial transfer as a multi-faceted therapeutic strategy for treating diseases

Through the delivery of functional mitochondria to impaired cells, MSCs can restore cellular energy production, enhance metabolic resilience, and counteract oxidative damage. This approach has shown potential in treating conditions such as neurodegenerative disorders, cardiovascular diseases, and immune-related disorders [26].

### Neurodegenerative and neurogenic disorders

**Fig. 4:**
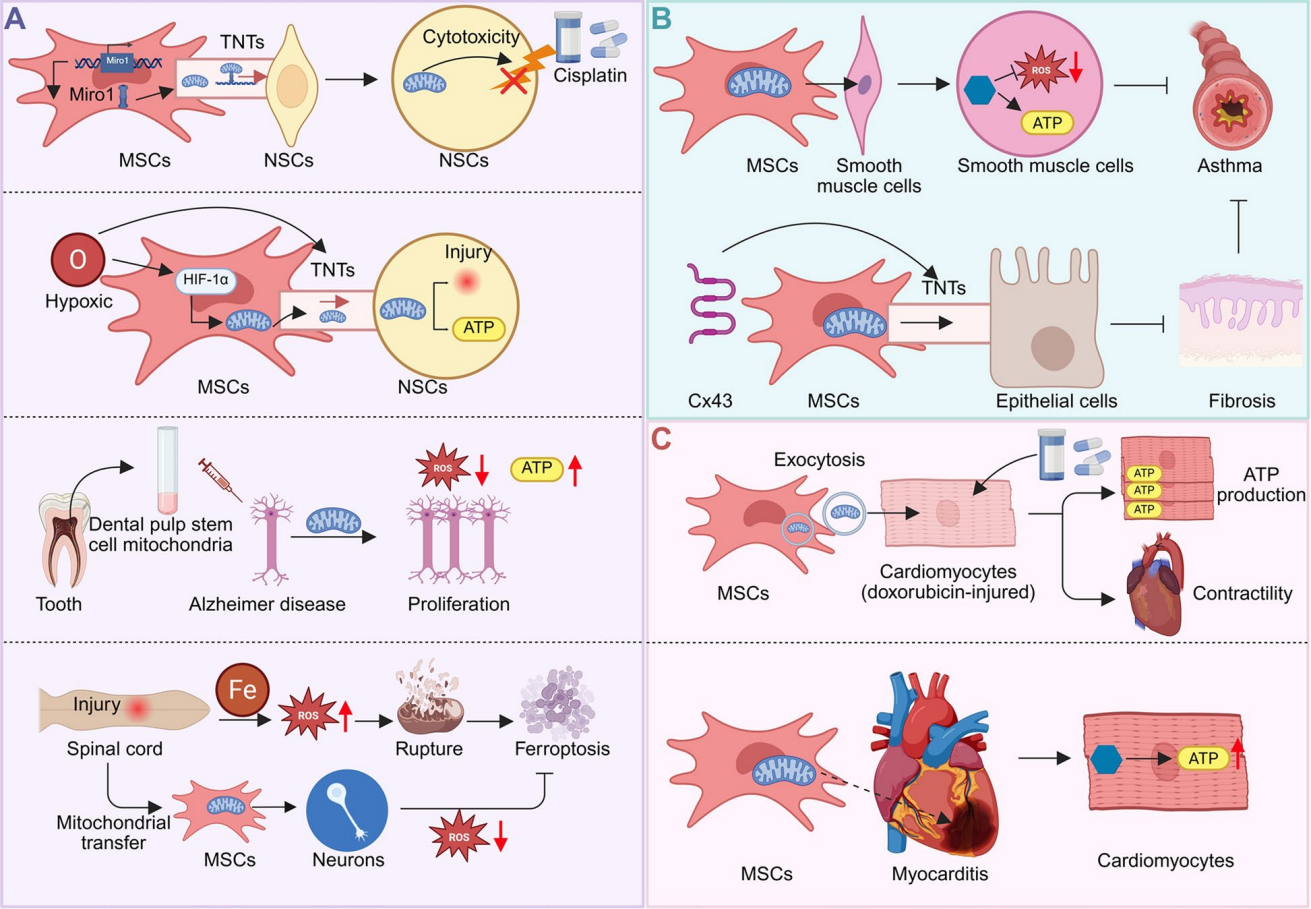
MSC-mediated mitochondrial transfer in neurological, pulmonary, and cardiovascular diseases. **A** In neurological disorders, MSCs transfer mitochondria to neural cells under stress or injury, restoring ATP production, reducing ROS, and preventing cell death. This includes models of cisplatin-induced toxicity, Alzheimer's disease, and spinal cord injury [28, 29, 31, 32]. **B** In pulmonary diseases, MSCs donate mitochondria to airway smooth muscle and epithelial cells, enhancing bioenergetics and limiting oxidative damage and fibrosis, with Cx43–mediated TNTs facilitating transfer [41]. **C** In cardiovascular injury, MSC-derived mitochondria improve ATP production and contractile function in damaged cardiomyocytes, highlighting their potential in myocardial repair [47]

Mitochondrial transfer from MSCs has emerged as a potent strategy for treating neurodegenerative and neurological diseases by preserving neuronal function and promoting regeneration [27]. MSCs protect neural stem cells (NSCs) from cisplatin-induced toxicity by transferring healthy mitochondria via actin-based structures, an effect enhanced by Miro1 overexpression. This preserves mitochondrial function and NSC survival, and maintains neural progenitor populations in vivo, underscoring the role of mitochondrial donation in MSC-mediated neuroprotection [28] (Fig. 4A). Similarly, mitochondrial transfer from MSCs to stressed neurons improves survival and metabolism, while induced pluripotent stem cell-derived mesenchymal stem cells (iPSC-MSCs) mitigate hypoxia-induced neuronal damage in PC12 cells through TNT-mediated transfer, enhancing mitochondrial function and cell viability under stress [29] (Fig. 4A). In Parkinson’s disease (PD) models, human MSC-derived mitochondria reduce dopaminergic neuronal loss, suppress microglial activation, and improve motor function [30]. In AD models, mitochondria from dental pulp stem cells enhance neuronal proliferation, reduce oxidative stress, and mitigate hallmark AD pathologies such as Aβ and Tau aggregation [31] (Fig. 4A).

Beyond neurodegeneration, mitochondrial transfer shows therapeutic promise in neurogenic injuries. In spinal cord injury, mitochondrial quality control disruption promotes ferroptosis, while MSC-mediated transfer restores mitochondrial pools and inhibits ferroptosis by enhancing fusion [32] (Fig. 4A). Bone marrow mesenchymal stem cells (BMMSCs) improve recovery by transferring mitochondria to injured neurons via gap junctions, enhancing energy metabolism and reducing apoptosis, thus improving locomotor function [33]. CD157 enhances mitochondrial transfer from BMMSCs to injured neurons, promoting axon regeneration and functional recovery via Ca^2^⁺ signaling. In peripheral nerve injury, intraneural injection of BMMSC-derived mitochondria into sciatic nerves enhances axonal regeneration by inducing Atf3 and other regeneration-related genes in dorsal root ganglion neurons, facilitated by retrograde signaling and increased ROS and DNA breaks [34, 35].

Mitochondrial donation also plays a critical role in ischemic stroke recovery [36]. Umbilical cord-derived MSCs transfer mitochondria to neurons and ECs, reducing ROS and enhancing survival after ischemia–reperfusion injury, a process modulated by the host ROS response [37]. Similarly, MSCs restore mitochondrial activity in cerebral microvascular cells, promoting angiogenesis, reducing infarct size, and improving functional recovery [38].

### Respiratory diseases

MSC mitochondrial transfer is emerging as a promising treatment for respiratory diseases by enhancing mitochondrial function in lung cells, thereby mitigating oxidative stress and inflammation—key pathological drivers in conditions such as asthma, interstitial lung disease, and acute lung injury [39]. iPSC-MSCs alleviate oxidative stress-induced mitochondrial dysfunction in airway smooth muscle cells, reduce airway hyperresponsiveness, and dampen inflammation in mice through direct mitochondrial transfer [40]. Similarly, iPSC-MSCs transfer mitochondria to epithelial cells via TNTs, with Cx43 involvement, restoring mitochondrial function and reducing Th2 cytokines and asthma symptoms [41] (Fig. 4B). Embryonic stem cell-derived MSCs from Daewoong Pharmaceutical also demonstrate therapeutic potential in interstitial lung disease models by reducing fibrosis and inflammation, modulating immune responses, and improving mitochondrial function in alveolar epithelial cells [42].

In acute lung injury models, adipose-derived MSC exosomes transfer mitochondrial components to alveolar macrophages, restoring mitochondrial integrity, promoting oxidative phosphorylation, and shifting macrophages toward an anti-inflammatory phenotype, thus attenuating lung inflammation and maintaining immune homeostasis [43]. Likewise, MSCs transfer mitochondria to pulmonary microvascular ECs via TNTs, enhancing endothelial barrier integrity in sepsis-induced lung injury; inhibiting this process or downregulating TFAM in MSCs impairs barrier repair and worsens vascular permeability [44]. In acute respiratory distress syndrome (ARDS), mitochondrial transfer through extracellular vesicles promotes anti-inflammatory and phagocytic macrophage phenotypes, improves oxidative phosphorylation, and enhances outcomes in lung injury models. Furthermore, MSC-derived mitochondria enhance macrophage phagocytic activity, supporting antimicrobial responses critical in ARDS pathophysiology [45].

### Heart diseases

MSC mitochondrial transfer is an emerging therapeutic strategy for heart diseases [46]. Human adipose-derived MSCs transfer mitochondria to rat cardiomyocytes under hypoxic conditions, enhancing energy metabolism and improving cardiac function after myocardial infarction. This effect, associated with donor mitochondrial DNA detected in recipient myocardium, is critical for the therapeutic efficacy of MSC transplantation in ischemic cardiomyopathy [47] (Fig. 4C). Additionally, large extracellular vesicles from MSCs transfer mitochondria to doxorubicin-injured cardiomyocytes, improving cell viability, enhancing contractility and ATP production, and reducing oxidative stress [48].

### Kidney diseases

**Fig. 5:**
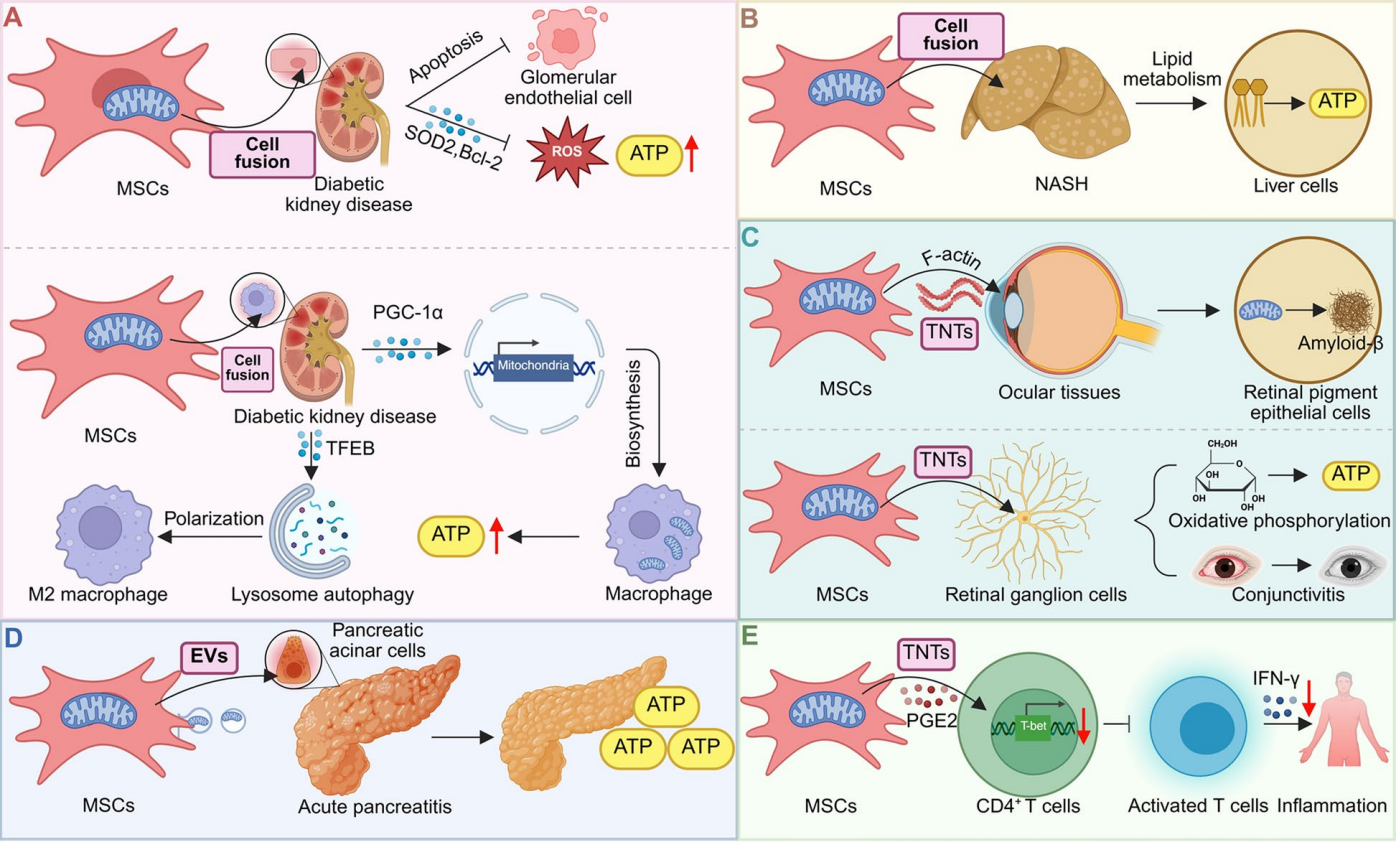
MSC-mediated mitochondrial transfer in metabolic, inflammatory, and degenerative diseases. **A** In diabetic kidney disease, MSCs transfer mitochondria to glomerular endothelial cells and modulate macrophage polarization via PGC-1α and TFEB signaling, enhancing ATP production and reducing inflammation [50]. **B** In NASH, MSC-derived mitochondria restore lipid metabolism and improve hepatic bioenergetics through direct fusion with hepatocytes [55]. **C** In retinal disorders, mitochondrial transfer to retinal pigment epithelial and ganglion cells reduces oxidative stress and supports visual function by enhancing mitochondrial activity [60]. **D** In acute pancreatitis, MSC-released extracellular vesicles deliver mitochondria to pancreatic cells, promoting energy recovery and reducing inflammatory damage [75]. **E** In immune-mediated conditions, mitochondrial transfer from MSCs to CD4⁺ T cells, along with PGE2 signaling, suppresses inflammatory cytokine production and modulates T cell responses [80]

MSC mitochondrial transfer provides a promising therapeutic strategy for renal disorders by promoting recovery and slowing disease progression [49]. For instance, MSCs rejuvenate injured glomerular ECs in diabetic kidney disease through mitochondrial transfer, enhancing mitochondrial function, reducing apoptosis, and lowering oxidative stress. In vivo, MSC treatment improved renal function, decreased fibrosis, and reduced oxidative damage, highlighting mitochondrial transfer as a key mechanism in MSC-mediated kidney repair [50] (Fig. 5A). MSC-derived mitochondria also enhance macrophage mitochondrial function, promoting M2 polarization through PGC-1α-driven biogenesis and TFEB-mediated lysosomal activation. Disruption of this mitochondrial transfer pathway abrogates MSC-induced anti-inflammatory effects and kidney protection [51] (Fig. 5A). Similarly, BMMSCs donate mitochondria to renal proximal tubular epithelial cells, reducing ROS and apoptosis while enhancing mitochondrial function and promoting renal repair in diabetic nephropathy [52]. Moreover, mitochondrial transfer to renal cortex cells mitigates doxorubicin-induced nephrotoxicity by decreasing oxidative stress, promoting tubular regeneration, and reversing renal dysfunction [53].

### Hepatic diseases

MSC mitochondrial transfer offers an innovative therapeutic strategy for liver diseases by enhancing mitochondrial activity and reducing inflammation [54]. Human MSCs alleviate high-fat-diet-induced non-alcoholic steatohepatitis (NASH) in mice by reducing hepatic lipid content and inflammation. This effect is attributed to the transfer of human mitochondria to mouse hepatocytes, enhancing oxidative capacity, promoting lipid breakdown, and restoring metabolic and tissue homeostasis [55] (Fig. 5B). Similarly, mitochondrial transfer from BMMSCs to hepatocytes improves liver function and metabolic regulation in a type 2 diabetes-associated non-alcoholic fatty liver disease (NAFLD) model by boosting mitochondrial activity, increasing ATP production, and reducing oxidative stress [56]. Additionally, extracellular vesicles from human umbilical cord-derived MSCs alleviate liver ischemia–reperfusion injury by delivering functional mitochondria to intrahepatic neutrophils. This restores neutrophil mitochondrial function, reduces NET formation, and improves liver recovery, highlighting the therapeutic potential of MSC-EVs in liver disease [57].

### Ocular diseases

MSCs transfer mitochondria to various ocular cells via F-actin-based TNTs, enhancing aerobic capacity and mitochondrial gene expression to support tissue repair and regeneration [58]. Transplantation of MSC-derived mitochondria into retinal pigment epithelial cells mitigates amyloid-beta-induced mitochondrial dysfunction and damage, improves tight junction integrity, and facilitates clearance of internalized amyloid-beta, offering a potential treatment for age-related macular degeneration [59]. Similarly, intravitreal injection of iPSC-derived MSCs in Ndufs4 knockout mice enhances retinal ganglion cell survival and function through mitochondrial donation, reducing dysfunction and inflammatory cytokine expression [60] (Fig. 5C). BMMSCs also transfer mitochondria to Müller cells via cell fusion and TNTs, improving mitochondrial function, reducing oxidative stress and gliosis, increasing mtDNA content, and promoting mitochondrial fusion to delay retinal degeneration [61]. Induced MSCs protect the ischemic retina by transferring mitochondria to immune cells, boosting oxygen consumption and ATP production, which supports CD4^+^CD25^+^ regulatory T cell (Treg) differentiation and Foxp3^ +^ Treg accumulation, thereby reducing inflammation and degeneration in ischemia–reperfusion injury [62]. Additionally, mitochondrial transfer via TNTs enhances corneal epithelial regeneration in dry eye disease, with the ROCK inhibitor Y-27632 increasing transfer efficiency through cytoskeletal remodeling [63]. MSCs also donate mitochondria to corneal epithelial cells under oxidative stress, enhancing survival and mitochondrial function via NFκB/TNFαip2 signaling, which improves wound healing in a rabbit model [64].

### Vascular diseases

MSC mitochondrial transfer offers an effective strategy for vascular-related disorders by restoring ECs function [65]. BMMSCs transfer mitochondria to cytarabine-stressed ECs via TNTs, mitigating apoptosis and enhancing EC proliferation and angiogenesis, suggesting potential in hematopoietic and vascular repair [66]. Transplanted MSC mitochondria also boost EC bioenergetics in ischemic tissue, enabling vessel formation independently of MSCs. This benefit is mediated through mitophagy activation via the PINK1-Parkin pathway, improving EC engraftment [65]. In diabetic wounds, impaired angiogenesis driven by NET-induced ferroptosis of ECs (via PI3K/AKT) is reversed by MSC-derived extracellular vesicles, which transfer functional mitochondria to neutrophils, reduce NET formation, and restore angiogenesis [67].

### Reproductive system disorders

Mitochondrial transfer from MSCs enhances cellular metabolism and function in reproductive disorders [68]. Urine-derived MSC mitochondria, physiologically similar to oocyte mitochondria, improve embryo development and metabolism in infertile females, particularly those of advanced age or with recurrent pregnancy failure, offering a non-invasive strategy without ethical concerns [69]. Similarly, adipose-derived MSC mitochondria improve oocyte quality and fertility in aged mice [70]. In males, MSC-derived mitochondria restore erectile function in a cavernous nerve injury model by enhancing smooth muscle content, reducing oxidative stress, and improving energy metabolism [71]. Additionally, stem Leydig cells enhance testosterone production and fertility through mitochondrial transfer to macrophages in testicular ischemia–reperfusion injury. This effect depends on ROS-mediated activation of TRPM7 channels, and TRPM7 knockdown impairs the therapeutic outcome [72].

### Inflammatory-related diseases

MSC mitochondrial transfer is a promising approach for modulating inflammation and promoting tissue repair [73, 74]. For instance, MSCs deliver functional mitochondria to damaged pancreatic acinar cells via extracellular vesicles, reversing metabolic dysfunction and maintaining ATP supply in acute pancreatitis. Hypoxia-preconditioned MSCs enhance mitochondrial membrane potential and reduce superoxide accumulation, facilitating metabolic remodeling [75] (Fig. 5D). Additionally, MSCs donate mitochondria to damaged tenocytes, enhancing mitochondrial function, reducing apoptosis, and promoting proliferation in Achilles tendinopathy models. This mitochondrial transfer is essential for decreasing inflammation and oxidative stress in tenocytes, improving tendon-specific markers, and remodeling tendon structure [76]. Interestingly, BMMSCs protect odontoblasts from pyroptosis in pulpitis by donating mitochondria through TNTs, reducing oxidative stress and NLRP3 inflammasome activity. Moreover, MSCs transfer mitochondria to stressed chondrocytes, improving their function and mitigating cartilage degradation in osteoarthritis (OA). This transfer, enhanced by mitochondrial dysfunction in chondrocytes and blocked by gap junction inhibitors, suggests a novel mechanism by which MSCs promote cartilage healing and highlights the therapeutic potential of MSC-based treatments for OA [77]. Similarly, mitochondrial transfer from BMMSCs to osteoarthritic chondrocytes enhances mitochondrial function, increasing membrane potential, respiratory chain activity, and ATP production. This transfer reduces chondrocyte apoptosis and promotes the secretion of type II collagen and proteoglycan [78].

### Immune-related disorders

Mitochondrial transfer from MSCs to immune cells represents a promising immunomodulatory therapy [79]. MSCs reduce Th1 responses by donating mitochondria to CD4⁺ T cells, lowering proliferation and IFN-γ production through T-bet downregulation, aided by prostaglandin E2 [80] (Fig. 5E). MSC mitochondria also enhance CD4⁺ T cell differentiation into Tregs and improve their suppressive function, reducing organ damage in GVHD models [74]. In rheumatoid arthritis, MSCs from healthy donors—but not RA patients—transfer mitochondria to Th17 cells, suppressing IL-17 and promoting Treg-like phenotypes [81]. UC-MSCs modulate T cell responses in SLE by transferring mitochondria and inhibiting autophagy, reducing apoptosis through suppression of mitochondrial biogenesis [82].

Additionally, MSCs stabilize FOXP3 expression and enhance Treg function via TNT-mediated mitochondrial transfer, involving BACH2 and SENP3 [83]. Both direct and indirect MSC-Treg contact enhances immunosuppression, dependent on HLA-C and HLA-DRB1 eplet compatibility, through co-transfer of mitochondria and membrane components [84].

### Genetic disorders

MSC-mediated mitochondrial transfer provides a potential therapy for genetic diseases involving mitochondrial dysfunction [85]. Wharton's jelly-derived mesenchymal stem cells (WJMSCs) show therapeutic potential in Duchenne muscular dystrophy by modulating WNT/TGF-β pathways and promoting mitochondrial transfer via the PI3K/Akt axis [86]. In mitochondrial encephalomyopathy, lactic acidosis, and stroke-like episodes (MELAS), WJMSCs overexpressing Miro1 enhance mitochondrial delivery to fibroblasts, improving intercellular communication, mitochondrial function, and reducing ROS and apoptosis [87]. In myoclonic epilepsy with ragged red fibers (MERRF) syndrome, WJMSCs selectively transfer mitochondria to defective cells, restoring mitochondrial activity and improving resistance to apoptosis [88]. In Leber's hereditary optic neuropathy (LHON), co-culturing MSCs with patient-derived neurons enhances mitochondrial recovery and metabolic function, suggesting therapeutic potential in mitochondrial optic neuropathies [89].

### Other diseases

MSC mitochondrial transfer extends to various other conditions. BMMSCs rescue nucleus pulposus cells from mitochondrial dysfunction and apoptosis via TNT-mediated transfer, dependent on Miro1 expression, supporting intervertebral disc regeneration [90]. Co-culture of MSCs with human islet β-cells enhances insulin secretion via mitochondrial transfer, especially under hypoxia, suggesting application in islet transplantation. In dexamethasone-induced muscle atrophy, UC-MSC mitochondria promote muscle regeneration by increasing muscle mass and desmin expression, and by inhibiting MAFbx and MuRF-1 via the AMPK-Akt-FoxO pathway [91]. Mitochondrial transfer between MSCs and chondrocytes also enhances proteoglycan deposition and DNA content in vitro, though donor mitochondria were not detectable in vivo after one year, suggesting a transient effect in cartilage repair [92].

Across diverse pathological contexts, MSC-mediated mitochondrial transfer has emerged not merely as a means of metabolic compensation but as a multi-dimensional biological intervention capable of reprogramming recipient cell fate, stress response, and intercellular communication. From neurons and cardiomyocytes to renal epithelial cells, macrophages, and T lymphocytes, mitochondria serve as functional cargos that influence cell survival, polarization, secretory phenotype, and even epigenetic memory. Notably, this transfer exhibits system-specific outcomes—such as ferroptosis suppression in neural injury, endothelial barrier restoration in sepsis, immune phenotype switching in ARDS and autoimmunity, and gamete rejuvenation in reproductive aging—highlighting a context-dependent precision that traditional paracrine models of MSC action fail to explain.

What distinguishes mitochondrial transfer from conventional cell therapy is its modularity and programmability. Mitochondria are not passive organelles; they are active regulators of intracellular signaling, redox tone, and metabolic hierarchy. Thus, the therapeutic potential of this mechanism depends not only on transfer efficiency but also on mitochondrial "quality," compatibility, and fate after internalization. The emerging evidence of mitochondrial control over immune checkpoint modulation, ferroptosis thresholds, and transcriptional landscapes suggests this process could be leveraged beyond tissue repair—to rewire pathological signaling networks in complex diseases. To unlock this potential, future studies should prioritize three directions: (1) systematic mapping of cell type–specific uptake mechanisms and mitochondrial processing pathways; (2) engineering MSCs or vesicle systems with enhanced mitochondrial bioenergetics, selectivity, and survival capacity; and (3) integrating organelle transfer into combinatorial therapies—such as immunotherapy, gene editing, or metabolic modulation.

## MSC mitochondrial transfer plays a context-dependent role in cancer treatment

While MSC-derived mitochondrial transfer has demonstrated promising therapeutic effects in metabolic, degenerative, and inflammatory diseases, its role in cancer is notably more complex and context-dependent. In contrast to other pathological settings where mitochondrial transfer supports cellular repair and recovery, in the tumor microenvironment, it may exert opposing effects—either promoting tumor progression or enhancing antitumor responses [16, 93]. Therefore, its application in oncology requires nuanced consideration.

Accumulating evidence suggests that MSCs can facilitate tumor growth and therapy resistance by donating functional mitochondria to cancer cells. In acute myeloid leukemia (AML), leukemic cells acquire mitochondria from MSCs via TNTs to evade oxidative phosphorylation inhibition, aided by increased mitochondrial fission and mitophagy that sustain cell survival and migration under stress [94]. Similarly, under chemotherapy-induced oxidative stress, MSCs in acute lymphoblastic leukemia (ALL) adopt a cancer-associated fibroblast phenotype and transfer mitochondria to ALL cells, promoting resistance [95]. In melanoma, tumor cells stimulate PGC1α-driven mitochondrial biogenesis in MSCs and subsequently acquire these mitochondria to enhance their proliferation and tumorigenicity [96]. In ovarian cancer, carcinoma-associated MSCs donate mitochondria to metabolically deficient tumor cells, enhancing chemoresistance and metastatic potential via transcriptional reprogramming and angiopoietin-like 3 (ANGPTL3) secretion, ultimately worsening prognosis [97].

Moreover, MSCs contribute to drug resistance across multiple cancer types [98]. For instance, mitochondrial transfer to prostate cancer cells enhances proliferation and counters cisplatin-induced cytotoxicity, though it increases vulnerability to ferroptosis [99]. In glioblastoma, MSC-derived mitochondria shift glioma stem cells from glycolysis to glutamine metabolism, conferring resistance to temozolomide (TMZ) and altering nucleotide synthesis pathways [100]. In multiple myeloma, tumor cells circumvent belantamab mafodotin–induced cytotoxicity by acquiring mitochondria from stromal cells, simultaneously gaining resistance to other agents like carfilzomib and venetoclax [101]. Furthermore, in T-ALL, mitochondrial transfer from tumor cells to MSCs promotes chemoresistance via ICAM-1–mediated adhesion and TNTs, suggesting a potential targetable axis [102].

Despite these protumorigenic effects, mitochondrial transfer can also potentiate antitumor immunity. In particular, the transfer of functional mitochondria from bone marrow stromal cells to exhausted CD8⁺ T cells restores their oxidative phosphorylation and spare respiratory capacity via Talin 2–dependent nanotube formation. These metabolically rejuvenated T cells demonstrate enhanced expansion, tumor infiltration, and reduced exhaustion, culminating in stronger antitumor activity and improved survival outcomes [103].

These findings underscore the dualistic nature of MSC-mediated mitochondrial transfer in cancer—capable of sustaining malignant cells or reinvigorating immune effectors depending on the recipient cell type and microenvironmental cues. Mitochondria in this context act as dynamic vectors of metabolic and transcriptional reprogramming rather than mere energy donors. Therapeutic strategies should thus move toward a context-specific, cell-targeted approach: suppressing mitochondrial donation to cancer cells while preserving or enhancing it toward immune cells. Such precision modulation of intercellular mitochondrial dynamics may open new avenues for reprogramming the tumor microenvironment and overcoming therapeutic resistance.

## Enhancing or suppressing MSC functions via mitochondrial transfer from diverse cell sources

Mitochondrial transfer to MSCs from various donor cells has emerged as a novel approach to modulate MSC biological functions. This bidirectional exchange not only enhances MSCs' regenerative properties but also expands their therapeutic scope by reprogramming their metabolism and signaling states [104]. Functional mitochondria transferred into BMMSCs have been shown to promote proliferation, migration, and osteogenic differentiation, partly through improved oxidative phosphorylation and ATP production, thereby facilitating bone regeneration [105]. Likewise, mitochondrial transfer into adipose-derived MSCs enhances their bioenergetics, reshapes their secretome, and improves tissue repair capacity in wound models [106]. Hematopoietic stem and progenitor cells can also restore impaired MSC functions by donating healthy mitochondria, which supports stromal niche recovery [107]. Among diverse donor sources, platelets represent an efficient and physiologically compatible mitochondrial reservoir. Platelet-derived mitochondria can restore mitochondrial dynamics and anti-apoptotic capacity in chemotherapy-damaged MSCs, and promote wound healing by activating de novo lipogenesis and enhancing pro-angiogenic responses under oxidative stress [108–110]. Conversely, mitochondrial transfer may exert detrimental effects depending on the donor cell context. In osteoporotic conditions, phenotypically altered macrophages deliver dysfunctional mitochondria to MSCs, inducing ROS accumulation and aberrant metabolic remodeling—specifically succinate buildup—which impairs osteogenic differentiation [111]. This pathological mitochondrial crosstalk reflects a metabolic hijacking process that disrupts stem cell homeostasis.

These findings underscore that mitochondrial transfer is not inherently beneficial; rather, its impact on MSCs critically depends on the metabolic fitness and immunological status of the donor cell. This highlights a fundamental concept: mitochondria function not merely as bioenergetic units, but as metabolically active signaling organelles capable of reprogramming the identity and functional state of recipient cells. Their ability to induce durable alterations in MSC behavior suggests that mitochondria may serve as vectors of epigenetic and metabolic memory, rather than acting solely as transient energy suppliers. Accordingly, future therapeutic strategies should emphasize the rigorous functional profiling and selective sourcing of donor mitochondria to ensure that transfer events consistently enhance, rather than compromise, MSC therapeutic efficacy.

## Strategies for enhancing mitochondrial transfer efficiency in MSCs

Due to the capacity of MSC mitochondrial transfer to modulate target cell functions and treat diseases, enhancing the efficiency of this transfer is crucial for maximizing therapeutic benefits. Recent advances have revealed that mitochondrial transfer efficiency is significantly influenced by the source of MSCs, their purification state, and external modulation strategies, each representing an important direction for therapeutic optimization.

MSC source and intrinsic properties play a fundamental role in determining mitochondrial transfer capacity and functional outcomes. MSCs derived from adipose tissue and bone marrow exhibit superior mitochondrial donation efficiency, whereas dental pulp and Wharton’s jelly MSCs, despite transferring fewer mitochondria, are more effective at attenuating oxidative stress in recipient cells [112]. Notably, human iPSC-MSCs demonstrate enhanced mitochondrial transfer capacity relative to BMMSCs. They efficiently rescue mitochondrial dysfunction in cigarette smoke-damaged airway epithelial cells [113] and show superior performance in cardiomyocyte rescue due to elevated MIRO1 and TNFαIP2 expression, which promote TNT-mediated transfer under TNF-α stimulation [114]. These findings suggest that source selection and intrinsic mitochondrial dynamics of MSCs should be key considerations in clinical application design. Conversely, MSCs from obese mice fed a high-fat diet exhibit impaired mitophagy and reduced cardiolipin content, resulting in diminished mitochondrial transfer capacity. Pharmacological intervention with pyrroloquinoline quinone restores cardiolipin levels and mitochondrial functionality, thereby enhancing intercellular transfer and alleviating disease features in allergic airway inflammation models [115]. These studies highlight the importance of metabolic health and donor condition in maintaining MSC functionality.

Cellular purification strategies also significantly influence mitochondrial transfer efficiency. Rapidly expanding clones (RECs) derived from MSCs exhibit superior mitochondrial donation capability compared to bulk MSC populations, markedly improving mitochondrial activity in deficient recipient cells [116]. Ultra-purified RECs enhance intercellular transfer through extracellular vesicles and Cx43-mediated gap junctions, representing a more precise and scalable therapeutic tool for mitochondrial diseases [117]. Furthermore, in a MELAS disease model, RECs showed greater capacity than conventional MSCs in donating mitochondria to iPSC-derived neurons, resulting in improved mitochondrial membrane potential, ATP/ROS balance, and oxygen consumption [118]. These findings support the refinement of MSC preparations as a technical strategy to improve treatment outcomes (Table 1).

**Table 1:** Impacts on MSC functions through mitochondrial transfer

Mitochondrial source	MSC type	Mechanism	MSC function	Therapeutic outcome	References
Donor BMMSCs	BMMSCs	Endocytosis	Enhanced MSC proliferation, migration and osteogenesis	Promoted bone defect repair	[105]
Y40-ADSCs	Y74-ADSCs	Endocytosis	Enhanced MSC proliferation, migration and differentiation	Improved tissue regeneration	[106]
Hematopoietic stem and progenitor cells	BMMSCs	Cell-contact mediated by Cx43	Enhanced MSC metabolism and regeneration	Increased hematopoietic engraftment success	[107]
Platelets	BA-MSCs	Endocytosis	Increased MSC apoptosis and suppressed cell cycle progression	Restored hematopoietic regulation	[110]
Platelets	Human MSCs	Clathrin-mediated endocytosis	Promoted metabolic remodeling, and pro-angiogenic activity	Enhanced wound healing efficacy	[109]
Platelet-rich plasma	Human MSCs	Extracellular vesicles, endocytosis	Stimulated MSC proliferation and pro-angiogenic properties	Promoted angiogenesis and wound healing	[108]
Macrophages with altered phenotypes	BMMSCs	Endocytosis, MVs, TNTs	Triggered ROS burst, and metabolic remodeling	Potential therapeutic target for osteoporosis treatment	[111]

Exogenous treatments and environmental modulation present another promising avenue for enhancing MSC-mediated mitochondrial transfer. For instance, pioglitazone combined with iron oxide nanoparticles enhances MSC mitochondrial biogenesis and intercellular transfer efficiency, which restores mitochondrial homeostasis in fibrotic lung models [119]. Iron oxide nanoparticles also promote Cx43-mediated junction formation, increasing transfer specificity and efficiency in pulmonary fibrosis without safety concerns [120]. Preconditioning MSCs with mitochondria from fetal cardiomyocytes enhances their bioenergetic profile via ROS signaling, improving cardiac function after transplantation in infarcted mouse hearts [121]. These results reveal the therapeutic value of using mitochondria themselves as biological modulators of MSC potency.

Moreover, biophysical cues such as matrix stiffness and autophagy status have been shown to regulate mitochondrial dynamics in MSCs. Cells cultured on softer matrices exhibit reduced mitochondrial function and osteogenic potential, which can be reversed by transferring mitochondria from cells cultured under higher stiffness, highlighting the relevance of physical environment in therapy design [122]. Autophagy modulation has also emerged as a key strategy for enhancing TNT formation and homotypic mitochondrial donation. Inducing autophagy lengthens TNTs and preserves mitochondrial membrane integrity, while inhibition disrupts transfer efficiency and regenerative signaling [123].

Several novel technical approaches have also shown promise. For example, extracorporeal shock wave treatment improves mitochondrial uptake in adipose-derived MSCs, increasing angiogenesis and cardiac function while reducing fibrosis and oxidative stress in dilated cardiomyopathy models [124]. Similarly, therapeutic hypothermia enhances MIRO1 expression and boosts mitochondrial transfer efficiency in ischemic stroke models, improving neuronal survival and long-term functional recovery [125]. A recently developed centrifugation-based method offers a generalized platform to enhance mitochondrial transfer across cell types, effectively restoring bioenergetic and oxidative parameters and reducing muscle atrophy (Table 2) [126].

**Table 2:** Strategies for enhancing mitochondrial transfer efficiency in MSCs

Specific strategy	Mechanism	Effects	Application	References
Cell source optimization	MIRO1 and TNFαIP2 expression ↑	Enhanced mitochondria transfer (iPSC-MSCs to CMs)	Rescued anthracycline-induced cardiomyocyte damage	[114]
Genetic modification	MIRO1 expression↑	Enhanced mitochondria transfer (MSCs to epithelial cells)	Boosted airway repair and reduced inflammation	[135]
Pre-treatment	MIRO1 expression↑	Mitochondrial transfer to lung cells, increased biogenesis	Mitigated pulmonary fibrosis	[119]
Extracellular vesicles	Mitochondrial function↑	Increased the ATP levels in brain ECs	Reducing oxidative stress and apoptosis	[136]
Purity level optimization	Mitochondrial purity↑	Enhanced mitochondrial functions	Potential neurological therapy	[118]
Matrix stiffness optimization	Matrix stiffness↑	Restored mitochondrial function	Reversing the osteogenesis ability of MSCs	[122]
Targeted delivery systems	Phosphatidylserine↑	Restored aerobic respiration and protection of ECs	Protected against ischemia–reperfusion injury	[134]
Hypothermia	MIRO1 expression↑	Improved mitochondrial transfer and long-term function	Reduced inflammation and apoptosis, and promoted neuronal viability neuroprotection after ischemic stroke	[137]
Centrifugation	AMPK/FoxO3/Atrogene pathway↓	Enhanced mitochondrial functions	Prevented muscle atrophy	[126]
Shock wave treatment	/	Enhanced mitochondrial delivery to adipose-derived MSCs	Promoted angiogenesis, reduced fibrosis, improved heart function	[124]
Preconditioning with fetal cardiac myocytes	ROS production↑	Enhanced mitochondrial degradation	Improved MSC repair, enhanced heart disease recovery	[121]

Collectively, these strategies—ranging from cell source optimization and purification protocols to preconditioning and biophysical modulation—represent a rapidly evolving technical foundation for improving the therapeutic utility of MSC-mediated mitochondrial transfer. Continued innovation in these areas will be critical for translating this promising biological mechanism into clinical success.

## Challenges and prospects

MSC-derived mitochondrial transfer offers significant therapeutic potential, yet its clinical application faces several key challenges that must be addressed for its successful implementation. One of the foremost challenges is the incomplete understanding of the underlying mechanisms that govern mitochondrial transfer [127]. While it is known that MSCs can transfer mitochondria through pathways such as TNTs and extracellular vesicles EVs, the prevailing view treats this behavior as a passive or reactive phenomenon in response to cellular injury. However, growing evidence suggests that mitochondrial donation by MSCs is an active, regulated biological response—possibly a conserved mechanism to restore metabolic homeostasis within damaged tissues. This raises new questions: what signals instruct MSCs to initiate transfer? How do they recognize which cells require support, and which mitochondria are suitable for donation? These questions remain largely unanswered.

In particular, the hypothesis that MSCs possess a quality control mechanism to selectively export functionally intact mitochondria has gained increasing traction. Rather than randomly releasing mitochondria, MSCs may utilize mitochondrial membrane potential, redox status, or post-translational modifications of mitochondrial proteins as indicators to sort and package high-performance organelles [128]. Regulatory pathways involved in mitochondrial homeostasis, such as PINK1/Parkin-mediated mitophagy or the mitochondrial unfolded protein response, may contribute to this selection process [128, 129]. Yet, direct evidence supporting such discriminatory sorting is limited, and mechanistic dissection through proteomics, mitochondrial reporters, and live-cell imaging is urgently needed. A clearer understanding of how MSCs discriminate “fit” mitochondria will be key to enhancing the safety and efficacy of this strategy.

Another critical challenge is the functional integrity of the transferred mitochondria. The therapeutic outcome is highly dependent on whether the delivered organelles are bioenergetically competent. Damaged or depolarized mitochondria may aggravate cellular dysfunction rather than ameliorate it [130, 131]. Thus, strict control over mitochondrial quality—not only in MSC cultures but also throughout the isolation, transfer, and delivery process—must be implemented in both research and clinical settings. Moreover, the long-term behavior of transferred mitochondria remains poorly understood. Do they integrate into host mitochondrial networks, persist transiently, or become degraded via host mitophagy pathways? Elucidating their fate will clarify the durability and limitations of mitochondrial transfer therapy.

Safety concerns, particularly immunogenicity, also require in-depth consideration. Although MSCs themselves are typically immune-evasive, mitochondria possess distinct immunostimulatory features such as unmethylated mtDNA and cardiolipin-rich membranes, which may activate pattern recognition receptors like TLR9 or the cGAS-STING pathway. In inflamed or allogeneic contexts, this could provoke innate immune responses or chronic inflammation [132]. Additionally, the risk of inadvertently enhancing tumor cell metabolism—thereby promoting progression or therapy resistance—cannot be ignored. Given that mitochondria play a central role in both energy metabolism and epigenetic regulation, their transfer may exert broader effects on recipient cell phenotypes than currently appreciated. Longitudinal studies in immunocompetent and tumor-bearing models will be essential to characterize these potential off-target risks.

Furthermore, the efficiency and specificity of mitochondrial delivery pose major translational obstacles. Most current approaches rely on the innate homing ability of MSCs, which is often insufficient for precise targeting. Moreover, the mechanical and biochemical barriers present in fibrotic, ischemic, or tumor tissues can impair both MSC migration and mitochondrial trafficking. To address this, future strategies should explore engineering MSCs with enhanced chemotactic receptors, or developing cell-free delivery platforms such as mitochondria-loaded nanoparticles or exosome-based carriers with tissue-specific ligands [133, 134]. Additionally, synthetic biology approaches may allow for the development of “programmable” MSCs that conditionally initiate mitochondrial transfer in response to defined pathological cues, increasing both efficacy and biosafety.

Beyond these mechanistic and technical hurdles, a more fundamental gap remains: what is the broader biological significance of MSC-mediated mitochondrial transfer? Rather than viewing it as a cell-autonomous repair mechanism, it may represent a higher-order, tissue-level coordination system in which MSCs serve as metabolic sentinels. By exporting mitochondria, they may actively reshape the bioenergetic landscape of injured or immunologically dysregulated environments, contributing to regeneration or immune modulation at the tissue level. This paradigm shift opens the door to repositioning mitochondrial transfer as a system-level therapeutic strategy, extending its application from acute injury repair to chronic disease modulation and tumor immunometabolic remodeling.

## Conclusion

In conclusion, MSC-derived mitochondrial transfer is emerging as a powerful therapeutic strategy, showing efficacy across a range of condition. By directly addressing mitochondrial dysfunction, this approach offers a targeted means to restore cellular energy and function. As the understanding of this process deepens and techniques improve, MSC mitochondrial transfer is poised to become a significant strategy in the treatment of complex diseases, bridging the gap between current therapies and the need for more effective interventions.

## Data Availability

Not applicable.

## Abbreviations

AD: Alzheimer’s disease
AIFM1: Apoptosis inducing factor mitochondrion associated 1
Akt: Protein kinase B
ALL: Acute lymphoblastic leukemia
AML: Acute myeloid leukemia
AMPK: AMP-activated protein kinase
ANGPTL3: Angiopoietin-like 3
ARDS: Acute respiratory distress syndrome
ATP: Adenosine triphosphate
Bcl-2: B-cell lymphoma 2
BBB: Blood–brain barrier
BMMSC: Bone marrow mesenchymal stem cell
Cx43: Connexin 43
DNA: Deoxyribonucleic acid
ETC: Electron transport chain
FoxO: Forkhead box O
Foxp3: Forkhead box P3
HIF-1α: Hypoxia-inducible factor 1-alpha
HLA: Human leukocyte antigen
iPSC-MSC: Induced pluripotent stem cell-derived mesenchymal stem cell
IFN-γ: Interferon-gamma
IL-17: Interleukin 17
KEAP1: Kelch-like ECH-associated protein 1
LHON: Leber's hereditary optic neuropathy
MAFbx: Muscle Atrophy F-box
MDR: Multi-drug resistance
MELAS: Mitochondrial encephalomyopathy, lactic acidosis, and stroke-like episodes
MERRF: Myoclonic epilepsy with ragged red fibers
NAFLD: Non-alcoholic fatty liver disease
NASH: Non-alcoholic steatohepatitis
NF-κB: Nuclear factor-κB
NSC: Neural stem cell
OA: Osteoarthritis
PD: Parkinson’s disease
PGAM5: Phosphoglycerate mutase family member 5
PGC-1α: Peroxisome proliferator-activated receptor gamma coactivator 1-alpha
PI3K: Phosphatidylinositol-3-kinase
REC: Rapidly expanding clone
ROS: Reactive oxygen species
Sig-1R: Sigma-1 receptor
SOD2: Superoxide dismutase 2, mitochondrial
Treg: Regulatory T cell
TGF-β: Transforming growth factor-beta
TMZ: Temozolomide
TNF-α: Tumor necrosis factor alpha
TNFαIP2: Tumor necrosis factor alpha induced protein 2
TNT: Tunneling nanotube
TRPM7: Transient receptor potential melastatin 7
WJMSC: Wharton's jelly-derived mesenchymal stem cell
